# Exercise as a Therapeutic Intervention for Chronic Disease Management: A Comprehensive Review

**DOI:** 10.7759/cureus.74165

**Published:** 2024-11-21

**Authors:** Maryam Fairag, Saif A Alzahrani, Naif Alshehri, Arjwan O Alamoudi, Yazeed Alkheriji, Omar A Alzahrani, Abdulrahman M Alomari, Yahya A Alzahrani, Shahad Mohammed Alghamdi, Amer Fayraq

**Affiliations:** 1 Family Medicine, Makkah Healthcare Cluster, Makkah, SAU; 2 Preventive Medicine, King Abdulaziz Medical City, Jeddah, SAU; 3 Public Health, Ministry of Health, Riyadh, SAU; 4 Emergency, Khalis General Hospital, Makkah, SAU; 5 College of Medicine, King Saud Bin Abdulaziz University for Health Sciences, Jeddah, SAU; 6 College of Medicine, AlBaha University, AlBaha, SAU; 7 Preventive Medicine, King Abdullah International Medical Research Center, Jeddah, SAU

**Keywords:** chronic disease management, exercise therapy, physiological mechanisms, quality of life, therapeutic intervention

## Abstract

This comprehensive review examines exercise as a therapeutic intervention for managing chronic diseases. It explores the physiological mechanisms behind physical activity's beneficial effects and its impact on various conditions, including cardiovascular disease, diabetes mellitus, obesity, and mental health disorders. Drawing from current literature and research findings, this review highlights how regular exercise significantly reduces mortality rates, improves disease outcomes, and enhances the overall quality of life for those with chronic illnesses. It discusses specific exercise recommendations for different conditions, emphasizing the importance of tailored physical activity programs. The review also addresses exercise's potential as a cost-effective and accessible treatment option, which may complement or, in some cases, reduce the need for pharmacological interventions. Ultimately, this review aims to equip healthcare professionals with a thorough understanding of exercise's therapeutic potential in chronic disease management, supporting the integration of physical activity into comprehensive treatment plans.

## Introduction and background

Physical activity has deep roots in ancient history. Around 3000 B.C., during the early Bronze Age, it is believed that the Indus Valley civilization laid the groundwork for what we now know as yoga [1]. The World Health Organization (WHO) defines physical activity as any skeletal movement that has high energy expenditure [2]. It ranges from low intensity, such as walking, to high intensity, like cycling. Regular exercise has shown great benefits in reducing premature mortality as well as reducing the risk of more than 25 non-communicable diseases across all age groups [3]. Diabetes, cardiovascular disease, and cancer are the top causes of morbidity and mortality worldwide [4]. Although pharmacological options have demonstrated their efficacy in the management of these conditions, an active lifestyle remains an effective way to prevent and treat these conditions [2,4]. The WHO recommends that patients with chronic conditions do at least 150 minutes per week of moderate-intensity aerobic exercises or their equivalent [2]. For every increase in 1-metabolic equivalent (MET), there is a reduction in premature mortality of 10-25% [5]. Exercise also has a preventive role in breast, colorectal, lung, and prostate cancers and increases survival after diagnosis [2]. 

For individuals with diabetes or prediabetes, physical activity is shown to reduce glycosylated hemoglobin (HbA1C) levels, decrease insulin resistance, decrease lipid levels, increase insulin sensitivity, and improve blood pressure [2,6]. For those who have hypertension, exercise is both a preventive and therapeutic treatment; it can reduce systolic blood pressure by 12 mm Hg and diastolic by 6 mm Hg, and improve cardiovascular function. Other effects include improvements in mental health, sleep quality, academic performance, and reduced incidence of depression [2]. On the other hand, a sedentary lifestyle or physical inactivity refers to acts that are done in a sitting or reclined position, e.g., watching TV or playing video games, that have low energy expenditure, i.e., less than 1.5 METs [3]. A paper published in 2024 states that globally, around one-third (31.3%) of the adult population was physically inactive [7]. It also showed that females were less active than males, and adults older than 60 years were less active than younger adults. Physical inactivity has shown an increase of up to 30% in mortality compared to active groups. It is estimated that physical inactivity costs globally up to $27 billion each year if it does not decrease [2]. People with a sedentary lifestyle are associated with an increased risk of chronic disease and premature mortality [3].

This review aims to provide a comprehensive examination of the role of exercise in the management of chronic diseases. It will explore the physiological mechanisms underlying the therapeutic effects of exercise and discuss its impact on specific chronic conditions, including cardiovascular disease, diabetes mellitus, obesity, and mental health disorders. The inclusion criteria for papers in this review were peer-reviewed articles published in English, studies focusing on the effects of exercise on chronic diseases (including cardiovascular disease, diabetes mellitus, obesity, and mental health disorders), and both original research articles and systematic reviews, with preference given to more recent studies. Exclusion criteria included non-English language publications, studies focusing solely on acute conditions or injuries, and case reports and opinion pieces.

## Review

Physiological basis of exercise and chronic disease

Exercise has a significant benefit for many individuals, particularly those diagnosed with chronic diseases, as it affects their lives in various aspects. For instance, studies have demonstrated that moderate-intensity exercise positively impacts individuals with end-stage kidney disease by enhancing physical function, both during and outside dialysis [8]. Additionally, research has shown that resistance training significantly enhances the levels of inflammatory markers in individuals with chronic kidney disease [9]. In addition, exercise therapy has a fundamental role for individuals with rheumatoid arthritis, even with the variability of the type, extremity, and duration of the exercise, since extending the duration of physical activity can efficiently reduce the severity of rheumatoid arthritis [10]. Furthermore, regarding individuals with type 2 diabetes (T2D), moderate-to-high-intensity aerobic exercise is recommended in order to preserve an optimal level of HbA1c, total cholesterol (TC), triglycerides (TG), and high-density lipoprotein (HDL), as well as the capability to reduce body weight [11]. Moreover, since many individuals nearing retirement age suffer from obesity and loss of muscle mass, the best way to maintain a healthy lifestyle is to follow a combination program including energy constraints and resistance exercise [12]. A randomized trial study found that six weeks of aerobic exercise contributed to diminishing serum-free fatty acids, blood sugar, and insulin resistance [13]. 

The pancreas produces the hormone insulin, which regulates blood sugar and the body’s energy. On the other hand, insulin resistance results when the body does not respond to this hormone, which consequently leads to diabetes, hypertension, hyperuricemia, and metabolic syndrome if left untreated [14]. Because insulin resistance is linked to an abnormal buildup of free fatty acids in adipose tissue, lipotoxicity, endoplasmic reticulum stress that is not controlled properly, and mitochondrial dysfunction, exercise can greatly reduce the risk of insulin resistance by reducing visceral fat tissue. Additionally, the excess adipose tissue significantly contributes to the disruption of the xanthine oxidoreductase enzyme. This leads to issues with the production of uric acid, subsequently impacting the insulin resistance that overweight or obese children and teens experience. The indices of insulin resistance and metabolic and non-metabolic processes can dramatically change after practicing aerobic exercises, which favorably lower oxidative stress and inflammatory adipokines [14]. Also, premenopausal women who enroll in short-term aerobic exercise can observe a substantial modification and improvement in their glucose and lipid metabolism, even if there is no significant change in body weight [13]. 

Exercise can not only improve metabolic functions but also play an essential role in cardiovascular outcomes. Aerobic exercise enhances both the functions of the coronary artery and vascular vasomotion [15]. Exercise can noticeably induce cardiovascular adaptation by integrating systemic, cellular, and molecular signaling pathways. This adaptation takes place during both acute and long-term physical exercise, which leads to cardiac growth by increasing the size of myocytes. Therefore, individuals with cardiovascular diseases require significant physical activity to achieve remarkable health modifications, such as reducing mortality risks [16]. Moreover, exercise can significantly affect revascularization, hence strengthening the cardiovascular system by enhancing exosomes. Research has shown that in order to release exosomes and regulate the expression of miRNAs and proteins that prevent myocardial apoptosis, lower myocardial infarction volume, boost angiogenesis, enhance microvessel density, eliminate myocardial fibrosis, and preserve endothelial cells, moderate exercise is an important choice [17]. To sum up, physical exercise has a major impact on different parts of the body including the urinary, rheumatology, and cardiovascular systems. Individuals need to introduce exercise in their lifestyle in order to promote their health.

Exercise and cardiovascular disease

One of the most effective aspects of cardiovascular health is physical activity. Physical activity can decrease the incidence of cardiovascular diseases, such as stroke and heart failure, slow down the progression of cardiovascular diseases, and reduce the mortality rates associated with cardiovascular disease [18]. The level of physical activity that leads to positive outcomes in terms of cardiovascular health and fitness is comparable for men and women of all ages, including the elderly, and for adults of different racial and ethnic backgrounds. Regular physical activity has a well-established favorable correlation with overall cardiovascular health [19]. Regular exercise that meets or exceeds physical activity guidelines reduces cardiovascular risk and mortality and plays an important role in primary and secondary cardiovascular disease prevention [20]. The most recent recommendations from the WHO regarding physical activity for health benefits suggest that adults aged 18 to 64 should engage in 150 to 300 minutes of moderate-intensity aerobic exercise each week, 75 to 150 minutes of vigorous-intensity aerobic exercise, or a combination of both [21]. 

It is also advised to perform muscle-strengthening exercises involving all major muscle groups at a moderate or greater intensity on two days per week. For individuals above 65 years, the physical activity guidelines are similar, but they should also engage in multicomponent exercises focusing on functional balance and strength training at a moderate or greater intensity for three days per week [21]. Following the WHO physical activity guidelines has been associated with a 23% to 40% and 27% to 31% reduction in CVD [22-24], and all-cause mortality [25,26]. The amount of weekly physical activity is inversely related to the reduction of cardiovascular disease risk. The most notable benefits are seen when transitioning from being inactive to engaging in 30 minutes of daily walking [27]. Engaging in aerobic exercise at moderate or vigorous intensity, as recommended by current guidelines, greatly decreases the risk of cardiovascular disease and overall mortality [27]. However, higher-intensity exercise may provide additional cardiovascular benefits and reduce risk compared to moderate-intensity aerobic exercise [28].

Exercise and diabetes mellitus

Lifestyle modifications have always been the first choice in many chronic diseases such as T2D, gestational diabetes mellitus (GDM), hypertension, obesity, irritable bowel syndrome, chronic obstructive pulmonary diseases, and many more. One of the most essential lifestyle modifications is exercise, and it has a profound impact on various chronic diseases such as DM and GDM [29]. Studies have shown that exercise is an effective intervention for both DM and GDM in which exercise can significantly decrease blood sugar, making the body’s cells more sensitive to insulin, thus decreasing the risk and complications of DM [30]. Exercise is a great and effective method to control glucose in GDM patients and even can delay or reduce the use of insulin. Furthermore, exercise allows cells to use glucose more efficiently due to improving insulin sensitivity [31]. Exercise can help with weight management, and maintaining a healthy weight is important in managing DM; it promotes healthy weight gain in pregnancy and reduces the risks of GDM. Exercise reduces the risk of cardiovascular diseases, neuropathy, and retinopathy which are common in DM, Also, exercise regularly during pregnancy decreases the risk of pregnancy-related complications and cesarean delivery [30]. 

Exercise improves the overall mental well-being of DM and GDM patients by reducing stress and anxiety [31]. Aerobic exercise and strength training are recommended and beneficial, and the aim is moderate-intensity exercises for 150 minutes per week [30,31]. Exercise regimens should always be incorporated in the treatment plan for DM and GDM as it is a cornerstone of lifestyle modification. Each patient with a specific exercise plan, and it is also cost effective in a way to prevent the patient from going into medication and added costs. Evidence-based medicine proved that exercise is profoundly beneficial to the patient and the outcome of the disease [30]. Also, patient education plays a key role in emphasizing the importance of exercise for the patient. Exercise is an important and powerful intervention for DM and GDM in which it can improve function, quality of life, disease outcome, and overall health [32]. 

Picard et al., in their systematic review, reported that following exercise training, all heart rate variability (HRV) measures showed improvement [33]. There was a notable increase in the standard deviation of normal-to-normal intervals (SDNN) (effect size 0.59, 95% confidence interval {CI} 0.26-0.93). High-frequency (HF) power also rose (0.58, -0.16-0.99), while both low-frequency (LF) power (-0.37, -0.69 to -0.05) and the LF/HF ratio (-0.52, -0.79 to -0.24) saw reductions. In contrast, the control group experienced no significant changes [33]. Some studies have suggested that aerobic exercises are better for the management of DM. For example, a systematic review and meta-analysis reported that after the exercise interventions, aerobic exercise led to a larger drop in Hb1AC levels compared to resistance exercise. The difference was 0.18% (1.97 mmol/mol), with a 95% confidence interval ranging from 0.01 to 0.36 [34]. Similarly, a systematic review and meta-analysis by Jang et al. reported that compared to the control group, the exercise intervention across 17 studies resulted in a notable decrease in HbA1c levels, with a weighted mean difference (WMD) of -0.58% (95% CI: -0.89 to -0.27; I² = 73%) [35]. While the intervention did not lead to significant changes in overall body weight, a subgroup analysis of eight aerobic training studies did reveal a meaningful weight reduction of 2.25 kg (WMD: -2.25 kg; 95% CI: -4.36 to -0.13; I² = 17%) [35].

Some studies have documented that high-intensity exercises are more effective compared to low-intensity exercises for DM. Feng et al., in their systematic review and meta-analysis, reported that compared to moderate-intensity aerobic exercise or traditional control methods, high-intensity interval training significantly improves various metabolic markers [11]. High-intensity training led to a notable reduction in fasting blood glucose (MD: -0.55; 95% CI: -0.85 to -0.25, Hedges’ g = 0.98), 2-hour post-glucose levels (MD: -0.36; 95% CI: -0.57 to -0.14, Hedges’ g = 1.05), and fasting insulin (MD: -0.41; 95% CI: -0.79 to -0.03, Hedges’ g = 1.07). It also significantly lowered HbA1c (MD: -0.60; 95% CI: -0.84 to -0.36, Hedges’ g = 2.69), TC (MD: -0.58; 95% CI: -0.80 to -0.36, Hedges’ g = 2.36), TG (MD: -0.50; 95% CI: -0.86 to -0.14, Hedges’ g = 1.50), and LDL cholesterol (MD: -0.31; 95% CI: -0.56 to -0.08, Hedges’ g = 0.91). Additionally, high-intensity training boosted HDL cholesterol levels (MD: 0.62; 95% CI: 0.29 to 0.95, Hedges’ g = 1.19). All these effects are statistically significant, with p-values below 0.01 [11].

Exercise and obesity

Metabolic syndrome refers to a collection of metabolic disturbances that are associated with an increased risk of T2D and cardiovascular diseases. To be diagnosed with metabolic syndrome, an individual must exhibit three or more specific criteria: a waist circumference exceeding 40 inches in men and 35 inches in women, HDL cholesterol levels below 40 mg/dL for men and below 50 mg/dL for women, fasting glucose levels at or above 100 mg/dL, and blood pressure measurements of 130/85 mm Hg or higher [36]. In a 2023 study by Chou et al., it was found that both men and women experienced a significant reduction in their risk of developing metabolic syndrome, regardless of the quantity of physical exercise performed [37]. Another research conducted by Amirfaiz et al. identified a clear association between a sedentary lifestyle and the incidence of metabolic syndrome [38]. This study emphasized that lower levels of physical activity correlate negatively with the risk of developing the condition, suggesting that increasing exercise could help lower these risks [38]. Additionally, a study by Kadgoglou et al. provided further evidence supporting the role of physical activity [39]. It discovered that intense exercise, particularly resistance training, was linked to an increase in the levels of active muscle GLUT4. This protein is vital for the insulin-regulated uptake of glucose in muscle cells, which can lead to improved lipid profiles. Therefore, engaging in regular physical activity is not only beneficial for reducing the risk factors associated with metabolic syndrome but also plays a significant role in enhancing overall metabolic health, particularly in terms of glucose regulation and lipid management [39].

A systematic review investigated the effect of different types of regular exercise on physical fitness in adults with overweight or obesity. When comparing different types of exercise, resistance training showed less improvement in VO2max compared to aerobic exercise. High-intensity interval training, however, proved to be a bit more effective than traditional aerobic workouts. Interestingly, combining aerobic and resistance exercises did not show any added benefit for VO2max compared to aerobic exercise alone. For boosting muscle strength, though, adding resistance training to the routine was recommended [40]. Another systematic review and meta-analysis revealed that engaging in regular aerobic exercise led to a notable reduction in waist circumference, averaging a decrease of 3.2 cm (with a 95% CI of −3.86 to −2.51, p ≤ 0.001) compared to those in the control group. This change in waist size was closely linked to a reduction in visceral adipose tissue, showing a strong correlation (β = 4.02; 95% CI 1.37 to 6.66, p = 0.004). Interestingly, vigorous-intensity workouts resulted in an even greater decrease in waist circumference, averaging −4.2 cm (95% CI −4.99 to −3.42, p < 0.0001), while moderate-intensity exercises led to a reduction of −2.50 cm (95% CI −3.22 to −1.79, p = 0.058)[41].

Some studies have also reported improvement in psychological outcomes in obese patients [42]. Exercise led to significant improvements in quality of life (QoL), particularly in physical health, but it did not have a noticeable impact on reducing depression. Participants experienced large effect sizes in the physical aspects of their well-being, and there were also enhancements in vitality and mental health. Additionally, levels of exercise self-efficacy and autonomous motivation saw consistent improvements, reflecting a more positive outlook on engaging in physical activity [42]. An overview of 12 systematic reviews and 149 studies revealed that exercise resulted in notable weight loss, with four systematic reviews and meta-analyses reporting MD between −1.5 kg and −3.5 kg [43]. Participants also experienced reductions in fat showing MDs from −1.3 kg to −2.6 kg. Additionally, visceral fat loss was observed in three systematic reviews, with SMD ranging from −0.33 to −0.56. Importantly, there was no significant difference in weight, fat, or visceral fat loss when comparing aerobic exercise to high-intensity interval training, provided that the energy expenditure was equivalent [43].

Exercise and mental health 

Mental disorders affect a significant portion of the global population, with around 12% experiencing some form of mental illness in 2019. These conditions are not only common but also profoundly impactful, accounting for about 5% of all disability-adjusted life years (DALYs) and 16% of the years people live with disability worldwide [44]. When it comes to treatment, medications like antidepressants [45] and antipsychotics [46] are often the first lines of defense. Yet, their long-term benefits are increasingly being questioned. For many, these drugs do not lead to substantial, lasting relief, and they come with some challenging side effects, including considerable weight gain, higher blood sugar levels, and decreased sexual interest [47,48]. These issues can cause people to stop taking their medication, adding another layer of distress and disrupting their lives [49]. The connection between mental and physical health has been acknowledged for centuries, echoing the old saying, “a healthy mind in a healthy body.” This relationship is more than just a theory; it is a lived reality for many, where the struggle to manage both mental and physical well-being is a daily challenge [50].

Many studies have shown that being less physically active or spending more time sitting is linked to a higher risk of mental health struggles. In a recent survey of 1.2 million U.S. adults, those who exercised regularly reported feeling mentally healthier than those who did not, even after adjusting for various personal and demographic factors [51]. Similar results have come up in research looking at specific mental health issues. For instance, a recent meta-analysis involving nearly 267,000 people found that higher levels of regular physical activity were linked to a lower risk of developing depression, with a significant reduction in odds (OR = 0.83, 95% CI 0.79-0.88) across all age groups [52]. Another meta-analysis, which focused on anxiety, found that being more active was associated with lower chances of experiencing anxiety symptoms (OR = 0.87, 95% CI 0.77-0.99) and anxiety disorders (OR = 0.66, 95% CI 0.53-0.83), based on data from over 80,000 participants [53]. Research increasingly supports the idea that staying active can help protect against mental health issues. The evidence suggests that the more physical activity people engage in, the lower their risk of experiencing mental health problems [54]. This trend holds true across different types of exercise, with a clear dose-response relationship, essentially, as the amount of physical activity goes up, mental health outcomes tend to improve. Even after accounting for factors like smoking (with an odds ratio of 0.67, indicating a 33% lower risk), the benefits of being active remain significant [55]. People with moderate or low fitness levels are at a 23% and 47% higher risk, respectively, of developing mental health issues compared to those who are highly fit. Interestingly, combining aerobic and resistance exercises seems to provide even greater protective effects [56].

Many studies show that exercise, especially aerobic workouts, can significantly help with depression and anxiety. In fact, the benefits of aerobic training are often similar to what people experience with traditional medications. Research indicates that aerobic exercise leads to noticeable improvements in mood compared to doing nothing at all (effect size: 1.24) or when matched against standard control conditions (effect size: 0.68). These improvements are on par with what’s seen in common therapy or medication treatments. However, the positive impact of exercise can fade over time. At follow-up, the benefits tend to be much smaller (effect size: 0.22) [57] and usually do not last unless people keep up with regular workouts after the initial treatment ends [58]. This shows that staying active is key to maintaining those mental health gains. Although research on resistance training for depression is less extensive, the available evidence indicates that it can help alleviate symptoms of depression, with an effect size of 0.66 (95% CI: 0.48-0.83) [59]. However, there is considerable variation across studies. Exercise has also been explored as a supplementary treatment alongside standard outpatient care, showing that it can significantly enhance treatment outcomes over 2 to 3 months, with an effect size of −0.79 (95% CI: −1.01 to −0.57) [60]. Similarly, a systematic review of eight meta-analyses reported that exercise interventions are effective for the management of depressive symptoms in patients of all age groups (effect sizes ranging from − 0.10 to − 0.81) [61].

Apart from depressive disorders, the impact of exercise has also been documented in other mental health disorders. For example, a systematic review found that aerobic exercise led to notable improvements in various cognitive functions compared to those who did not exercise. Specifically, it boosted overall cognitive abilities (SMD = 0.21), attention and alertness (SMD = 0.32), working memory (SMD = 0.27), and verbal learning skills (SMD = 0.30). These benefits were more pronounced with group-based activities (SMD = 0.28), workouts led by professional trainers (SMD = 0.27), and when individuals exercised for at least 90 minutes a week (SMD = 0.26) over a period of 12 weeks or more (SMD = 0.22)[62]. Similarly, exercise has also been shown to have positive effects in reducing Aβ deposition in the treatment of Alzheimer’s disease [63]. Björkman and Ekblom, in their systematic review also reported that exercise is effective for the treatment of post-traumatic stress disorder (PTSD) [64].

Exercise prescription

Exercise prescription is a systematic approach to designing physical activity programs that cater to the unique needs of each individual. An effective exercise prescription for chronic disease management incorporates several fundamental principles: frequency, intensity, time, and type. However, exercise is often overlooked and underutilized in favor of medications or surgeries [65,66]. Several factors contribute to this, including a lack of awareness about how effective exercise can be, both among healthcare providers and patients. Many clinicians may not have a clear understanding of what makes an exercise program effective or have sufficient training on how to incorporate exercise into treatment plans [67]. In a review of 137 non-drug interventions across 133 trials, 61% failed to provide enough information on how the exercise was conducted, such as specifics about the intensity and procedures, making it hard for practitioners to replicate these approaches in real-world settings. This gap in detail can hinder the practical application of exercise as a treatment option [68].

Several recommendations regarding the prescription of physical exercise for various chronic diseases have been reported. For example, patients with stable ischemic heart disease are encouraged to aim for 30 to 60 minutes of moderate aerobic exercise, like brisk walking, at least five days a week, ideally every day. To further boost their fitness and lower their risk, they should also try to be more active throughout the day. This can be as simple as taking short walking breaks at work, gardening, or doing household chores to stay moving [69]. Similarly, patients with hypertension are encouraged to get at least 30 minutes of moderate-intensity aerobic activity, such as walking, jogging, cycling, or swimming, 5 to 7 days a week, totaling at least 150 minutes per week. Along with aerobic exercise, dynamic resistance training is also recommended 2 to 3 days per week, though isometric exercises are not advised [70,71]. The 2020 European Society of Cardiology (ESC) Guidelines on sports cardiology and exercise for patients with cardiovascular conditions echo these suggestions [72]. Notably, for the first time, these guidelines mention the potential role of isometric resistance exercise in managing hypertension [72].

For people with type 2 diabetes, about 150 minutes of moderate-intensity aerobic activity each week, spread over three to five days is recommended. In the case of rigorous aerobic exercise, 75 minutes each week, spread over three to five sessions is advised. This should be combined with two to three weekly workout sessions of moderate to high-intensity strength training [73]. For patients with chronic obstructive pulmonary disease (COPD), it is recommended to participate in two to three sessions each week for a duration of eight to 12 weeks. In addition to these supervised sessions, aim for one to two extra sessions each week that can be done at home. Each session should last around 60 minutes [73].

Exercise effect on quality of life

The concept of QoL was initially introduced by American economist J. Calbraith in the 1950s [74]. Later on, the WHO described it as how people from various cultures and value systems perceive their lives. This definition emphasizes individuals’ goals, expectations, standards, and the living conditions that matter most to them [74]. The concept of QoL encompasses physical health, psychological state, social relationships, and the environment in which individuals live. Chronic brain disorders often lead to a lower quality of life, with many people experiencing symptoms like low mood, depression, heightened stress, and cognitive difficulties [75,76]. These issues are closely connected; depression and cognitive problems are key factors that impact quality of life, while depression itself can further worsen cognitive function [77]. These challenges can bring about serious consequences, including poor adherence to treatments, loss of daily independence, and even increased risk of death. Exercise therapy, however, offers a promising approach to improving quality of life, mood, and cognitive function across different conditions. For instance, in the case of stroke, physical activity has demonstrated positive effects on various symptoms and is now recommended as part of standard care [78].

Li et al., in their meta-analysis, reported that short-term aerobic exercise led to noticeable improvements in overall health, with a standardized mean difference (SMD) of 0.66 (95% CI: 0.05 to 1.30) [79]. It also played a role in boosting mental health (SMD = 0.38, 95% CrI: -0.05 to 0.81) and enhancing role functioning (SMD = 0.48, 95% CrI: -0.27 to 1.20), though the effects varied across different outcomes [79]. Similarly, in breast cancer survivors, physical activity significantly improved QoL and reduced anxiety [80]. The impact of physical activity is also documented in patients with T2D. For example, an RCT by Soleimani et al. reported that physical activity significantly improved QoL in patients with T2D [81]. Similarly, a systematic review and meta-analysis reported that exercise significantly improved QoL compared to the control group, with an SMD of 0.384 (95% CI, 0.257 to 0.512; P < 0.001). Aerobic exercise, whether performed alone (SMD, 0.475; 95% CI, 0.295 to 0.655; P < 0.001) or combined with resistance training (SMD, 0.363; 95% CI, 0.179 to 0.548; P < 0.001), led to noticeable QoL improvements. However, resistance training on its own did not show a significant impact [82].

Recommendations

Based on the extensive evidence presented, healthcare providers should consider incorporating exercise as a key component in the management of chronic diseases. It is recommended that exercise prescriptions be tailored to individual patient needs, considering factors such as disease type, severity, and overall health status. Healthcare systems should prioritize the integration of exercise programs into standard care protocols, potentially through the development of specialized exercise clinics or the inclusion of lifestyle medicine experts in multidisciplinary care teams. Additionally, there is a need for improved education and training for healthcare professionals on the benefits and implementation of exercise interventions. Public health initiatives should also focus on promoting the importance of physical activity in disease management and prevention.

Future research should focus on developing more precise, disease-specific exercise guidelines. Long-term studies are crucial to assess the sustained effects of exercise interventions on chronic disease outcomes and quality of life. Investigating the optimal combination of exercise types, intensities, and durations for various chronic conditions would provide valuable insights for clinical practice. Furthermore, exploring the synergistic effects of exercise with other therapeutic approaches, such as nutrition and psychological interventions, could lead to more comprehensive and effective treatment strategies.

Strengths

This comprehensive review synthesizes a broad range of evidence on the therapeutic effects of exercise across various chronic diseases, providing a holistic perspective on its potential as an intervention. The inclusion of multiple meta-analyses and systematic reviews strengthens the reliability of the findings. The review covers not only the physiological benefits of exercise but also its impact on quality of life and mental health, offering a multidimensional view of its therapeutic potential.

Limitations

Despite its promising findings, this review has several limitations. The diversity in study designs, exercise interventions, and outcome measures across the included studies makes direct comparisons challenging. Moreover, the effectiveness of exercise interventions may vary depending on factors such as adherence, which was not consistently reported. The review also did not thoroughly explore potential adverse effects or contraindications of exercise for specific patient populations, which are important considerations for clinical implementation.

## Conclusions

This comprehensive review underscores the significant therapeutic potential of exercise in managing chronic diseases. The evidence presented demonstrates that regular physical activity can lead to substantial improvements in physiological outcomes, mental health, and overall quality of life across various chronic conditions. From cardiovascular diseases to diabetes, and mental health disorders, exercise has shown promising results in alleviating symptoms, improving functional capacity, and enhancing patient well-being. The review also highlights the importance of tailored exercise prescriptions and the need for their integration into standard care protocols. While challenges in implementation and areas for further research remain, the overwhelming evidence supports the crucial role of exercise as a cornerstone in chronic disease management. Moving forward, a concerted effort from healthcare providers, policymakers, and researchers is essential to fully leverage the therapeutic benefits of exercise in improving patient outcomes and public health.
